# Overexpression of *PlPAT1*, a GRAS transcription factor from *Paeonia lactiflora*, enhances salt stress tolerance in transgenic tobacco

**DOI:** 10.1186/s12864-026-13204-w

**Published:** 2026-08-01

**Authors:** Jian Cai, Xuemei Zhang, Cong Yan, Chao Lu, Zhongxuan Fu, Yaowu Liu, Gang Li

**Affiliations:** 1 School of Biology and Food Engineering, Fuyang Normal University, Fuyang, Anhui China; 2 Anhui Huatuo Academy of Traditional Chinese Medicine, Bozhou Vocational and Technical College, Bozhou, Anhui China

**Keywords:** *Paeonia lactiflora*, GRAS transcription factor, Salt stress, Functional verification

## Abstract

**Background:**

Soil salinization poses a significant threat to agricultural productivity worldwide, including the cultivation of *Paeonia lactiflora*. Understanding the molecular mechanisms of salt stress response and identifying key salt-tolerant genes are essential for breeding resilient varieties. The GRAS family of transcription factors is known to regulate plant growth, development, and abiotic stress responses; however, its role in *P. lactiflora* remains poorly characterized.

**Results:**

In this study, we identified a novel GRAS transcription factor from *P. lactiflora*, designated *PlPAT1*, based on its homology to Arabidopsis GRAS members. Under salt stress, *PlPAT1* expression was up-regulated in leaves and stems but down-regulated in roots. Subcellular localization revealed that PlPAT1 protein localizes to both the nucleus and cytoplasm. Transgenic tobacco lines overexpressing *PlPAT1* exhibited enhanced salt tolerance, accompanied by increased chlorophyll content, elevated levels of soluble sugars and proteins, and higher activities of antioxidant enzymes (SOD, POD, CAT). In contrast, malondialdehyde (MDA) and hydrogen peroxide accumulation were significantly reduced compared to wild-type plants.

**Conclusion:**

Our results demonstrate that *PlPAT1* functions as a positive regulator of salt stress tolerance, likely through modulating osmotic balance and enhancing reactive oxygen species scavenging capacity. These findings highlight the potential of *PlPAT1* as a candidate gene for improving salt tolerance in plants and provide a genetic resource for the utilization of saline-alkali land.

**Supplementary Information:**

The online version contains supplementary material available at https://doi.org/10.1186/s12864-026-13204-w.

## Introduction

The growth of *Paeonia lactiflora* is frequently constrained by abiotic stresses, including drought, extreme temperatures, and soil salinity, which collectively impair plant productivity and threaten food security [1]. Soil salinization alone affects approximately 7% of global land area and 20% of irrigated agricultural land, representing a major challenge to sustainable crop production [2, 3]. In response, the development of salt-tolerant crop varieties through genetic engineering and phytoremediation has emerged as a promising strategy for the reclamation of saline-alkali soils [4]. A deeper understanding of the molecular mechanisms underlying salt stress response in *P. lactiflora*, particularly the identification of key functional genes, is therefore essential for breeding resilient varieties.

Salt stress exerts its detrimental effects through a multi-phase process. Initially, it induces osmotic stress, limiting water uptake and impairing plant growth [5, 6]. With prolonged exposure, ionic toxicity prevails as excessive ions such as Na⁺, Cl⁻, and SO₄²⁻ accumulate, leading to membrane depolarization, nutrient imbalance, and cellular damage [7, 8]. Salt stress also suppresses the expression of cell cycle-related genes, further stunting plant growth [3], and can trigger notable morphological alterations, as observed in Portulaca oleracea, which exhibits reduced leaf area, plant height, and root length under high salinity [9].

To mitigate salt stress, plants activate a suite of physiological and biochemical adaptations.These include the synthesis and accumulation of osmoprotectants such as proline, betaine, soluble sugars, and proteins, which aid in osmotic adjustment, cellular structure stabilization, and enzyme protection [10–13]. Soluble sugars, including glucose, fructose, and trehalose, contribute not only to osmoregulation but also to redox homeostasis and reactive oxygen species (ROS) scavenging [14–16].

Concurrently, plants enhance their antioxidant systems to counteract ROS-induced oxidative damage. This system comprises both enzymatic components—such as superoxide dismutase (SOD), peroxidase (POD), and catalase (CAT)—which collaboratively neutralize superoxide anions and hydrogen peroxide, and non-enzymatic antioxidants like ascorbic acid and glutathione, which help maintain ROS balance [17–19]. Elevated activities of SOD, POD, and CAT are closely associated with improved salt tolerance in species such as rice, wheat, and sweet potato [20–22].

Transcription factors (TFs) play a central role in coordinating these complex stress responses. By binding to cis-elements in target gene promoters, TFs regulate the expression of downstream genes involved in abiotic stress tolerance [23, 24]. Functional studies using knockout, silencing, and overexpression approaches have confirmed that TF families such as bZIP, MYB, WRKY, NAC, and GRAS are critical in mediating plant adaptation to salinity, drought, and cold [25, 26]. Among them, GRAS proteins constitute a major plant-specific TF family, participating in diverse signaling pathways related to growth, development, and stress responses [27, 28].

The GRAS family has been extensively classified, initially into eight subfamilies—including SCR, DELLA, PAT1, and HAM—with recent genomic studies expanding this to include several new subgroups such as LAS, DLT, and NSP1/2 [29, 30]. GRAS genes have been identified in over 50 plant species, ranging from dozens to hundreds of members per species, illustrating their broad functional diversity and evolutionary significance [31–33]. Notably, numerous GRAS members have been implicated in abiotic stress tolerance. For example, SlGRAS40 enhances drought and salt tolerance in tomato [32], BoGRAS is linked to heat resistance in cabbage [34], and HcSCL13, BpGRAS34, and HhGRAS14 have all been shown to improve salt tolerance in Arabidopsis, birch, and hibiscus, respectively [27, 35, 36].

Despite these advances, the role of the GRAS family, and particularly the PAT1 subfamily, remains largely unexplored in *P. lactiflora*. Given the agronomic and ecological importance of this species, and the expanding global challenge of soil salinization, identifying and characterizing key transcriptional regulators of salt tolerance is of critical importance. Therefore, this study aimed to identify and functionally characterize a novel PAT1-type GRAS gene, *PlPAT1*, from *P. lactiflora*. We analyzed its expression patterns under salt stress, determined its subcellular localization, and validated its role in salt tolerance through heterologous overexpression in transgenic tobacco. Our findings provide the first functional insights into the PAT1 subfamily in *P. lactiflora* and highlight *PlPAT1* as a promising candidate gene for improving salt stress resilience in plants.

## Materials and methods

### Plant materials and culture

The experimental material was obtained from the *P. lactiflora* base in the laboratory. The biennial “Qihualushuang” variety with similar growth conditions was transplanted into a permeable plastic basin, retaining some of the original soil. The basin was filled with a mixed substrate consisting of nutrient soil and vermiculite in a 7:3 volume ratio. After watering, the pots were placed in the backlight of an open balcony for 10 days, then transferred to an artificial climate chamber. The conditions were set to 24 °C, 6000 lx light intensity, a 16 h light/8 h dark cycle, and a relative humidity of 50–60% for pot culture.

### Salt treatment methods

After the plants resumed growth, root, stem, and leaf tissues from those under normal conditions were collected as the 0 h control group. Following irrigation with a 300 mmol/L NaCl solution, tissues exposed to salt stress for 2 h, 4 h, 8 h, 12 h, and 24 h were sampled as experimental groups, with three biological replicates per treatment. Tissues were immediately weighed (0.1 g per sample), wrapped in aluminum foil, sealed, flash-frozen in liquid nitrogen, and stored at -80 °C.

### RNA extraction and quantitative real-time PCR analysis

Leaves of *P. lactiflora* subjected to salt stress for 0 h, 2 h, 8 h, 12 h, and 24 h were used as samples. Total RNA was extracted using the RNA extraction kit (Vazyme, Nanjing, China) according to the provided protocol. Reverse transcription was performed with the reverse transcription kit (Vazyme, Nanjing, China). RNA input was calculated to ensure equal total RNA across all reaction systems. gDNA wiper MIX was added prior to reverse transcription to remove residual genomic DNA. The actin gene (Plactin, GenBank accession: JN105299) was used as the reference gene. Gene-specific primers for qRT-PCR were designed using Primer 5.0 software and validated for specificity via NCBI Primer-BLAST. Three biological replicates were included, and all experiments were performed in triplicate.

### *PlPAT1* sequence cloning and bioinformatic analysis

Based on the qPCR results, Unigene13406, which is up-regulated under salt stress, was selected and named *PlPAT1* according to the phylogenetic tree constructed with this gene and Arabidopsis GRAS family genes. The CDS sequences of P. lactiflora were retrieved from the in-house transcriptome database, and the open reading frame (ORF) was identified using ORF Finder. Full-length primers for *PlPAT1* were designed using Primer 5.0 software. PCR amplification was performed with high-fidelity DNA polymerase (Vazyme, Nanjing, China) and cDNA from *P. lactiflora* leaves as the template. The cloned fragment was ligated into the pMD™19-T vector, transformed into E. coli DH5α, and sequenced by a biotechnology company.

The gene’s conserved domain was predicted using the NCBI CD-search tool, while the physicochemical properties of its encoded amino acids were analyzed with ProtParam.Protein hydrophobicity was analyzed via ProScale. Signal peptides were predicted using SignalP‑6.0. Transmembrane domains were predicted with TMHMM‑2.0. Protein secondary structure was predicted using SOPMA. Homology modeling of the tertiary structure was performed with SWISS‑MODEL. Subcellular localization was predicted using WOLF PSORT. Multiple sequence alignment was carried out using DNAMAN.The phylogenetic tree was constructed with MEGA11.

### Vector construction and tobacco transformation

The full coding sequence of *PlPAT1* was amplified using primers containing restriction enzyme sites for XbaI and SmaI, and then inserted into the expression vector pBI121 via multi-fragment recombination. The resulting vector was transformed into *E. coli* TOP10 competent cells, which were then screened on LB medium supplemented with 50 mg/L kanamycin (Kan). Single colonies were picked for plasmid extraction. The pBI121-*PlPAT1* vector was introduced into *Agrobacterium tumefaciens* GV3101 using the freeze-thaw method, and tobacco transformation was performed via the leaf disk method. Transgenic seedlings were selected on MS medium containing 50 mg/L Kan. Positive transgenic lines were transplanted after verification by RT-PCR.

### Culture and salt treatment of transgenic tobacco

Rooted transformed seedlings were removed from tissue culture flasks, and residual medium was gently rinsed off the roots with clean water to avoid damage. Damaged or aged leaves, as well as those near the root zone, were excised. Seedlings were transplanted into autoclaved substrate (peat soil: vermiculite = 1:1, sterilized at 121 °C for 20 min) with roots spread and covered to retain moisture. The plants were acclimated in a growth chamber at 20–22 °C under low light for 7 days. Prior to use, the substrate was soaked with water-soluble fertilizer: sterilized substrate was placed in pots on trays, filled with fertilizer solution until fully wetted. After acclimation, plants were cultured at 22 °C (5000 lx, 16 h light/8 h dark, 50–60% humidity) for 45 days, after which transgenic tobacco was ready for subsequent stress resistance assays.

Transgenic tobacco lines OE-2, OE-3, and OE-7, along with wild-type Ben’s tobacco at the same growth stage, were used as stress treatment subjects. Some leaves were collected before stress treatment as control samples. The plants were watered with a 300 mmol/L NaCl solution, and tobacco leaves were harvested after 7 days for physiological analysis.

### Subcellular localization

The full-length *PlPAT1* sequence was amplified using primers PlPAT1-RBF/RBR (Additional file 1: Table S1) with XbaI/BamHI restriction sites and cloned into the pCambia1300S-YFP vector via homologous recombination to generate the subcellular localization vector pCambia1300S-*PlPAT1*-YFP. This construct was transformed into Agrobacterium GV3101.

For infiltration, Agrobacterium cultures were pelleted, resuspended in infiltration buffer (10 mM MgCl2, 10 mM MES, pH 5.7, 150 µM acetosyringone), and adjusted to OD600 = 1.0. The suspension was infiltrated into pre-darkened tobacco leaves (1 day) using a 1 mL needleless syringe. Infiltrated plants were wrapped in black plastic bags, incubated in darkness for 12 h, then transferred to low-light conditions (2–3 days). Fluorescent protein expression was observed using confocal microscopy on leaf sections.

### Determination of physiological indexes of transgenic tobacco under salt stress

The chlorophyll content, MDA content, soluble sugars, soluble proteins, antioxidant enzyme activities, and hydrogen peroxide content in leaves of wild-type (WT) and transgenic tobacco plants, both under untreated and salt stress, were measured using assay kits from Jiangsu Keming Biotechnology Co., Ltd., China.

### Statistical analysis

Statistical analysis was performed using one-way ANOVA in SPSS 17.0 (SPSS Inc., USA), with Duncan’s Multiple Range test to identify significant differences between groups. Differences were considered significant at *p* < 0.05.

## Results

### Screening of the GRAS Gene Family and Identification of *PlPAT1* in *Paeonia lactiflora*

A total of 8 GRAS family unigenes were identified from our transcriptome database based on domain integrity and differential expression under salt stress. The identification criteria were as follows: (1) containing a complete GRAS domain verified by NCBI CD-search; (2) showing significant differential expression under 300 mmol/L NaCl treatment (*p* < 0.05); (3) possessing an intact open reading frame (ORF). According to phylogenetic relationships with *Arabidopsis thaliana* GRAS proteins, these unigenes were systematically named as: Unigene5852 (*PlDELLA1*, DELLA subfamily), Unigene13406 (*PlPAT1*, PAT1 subfamily), Unigene23140 (*PlSCL8*, PAT1 subfamily), Unigene25298 (PlSCL13, PAT1 subfamily), Unigene29920 (PlSCL1, PAT1 subfamily), Unigene33285 (PlSCL4, SCR subfamily), Unigene34122 (PlSCR1, SCR subfamily), and Unigene51998 (PlHAM1, HAM subfamily). Detailed information for these eight GRAS genes is listed in Table S1.

**Fig. 1:**
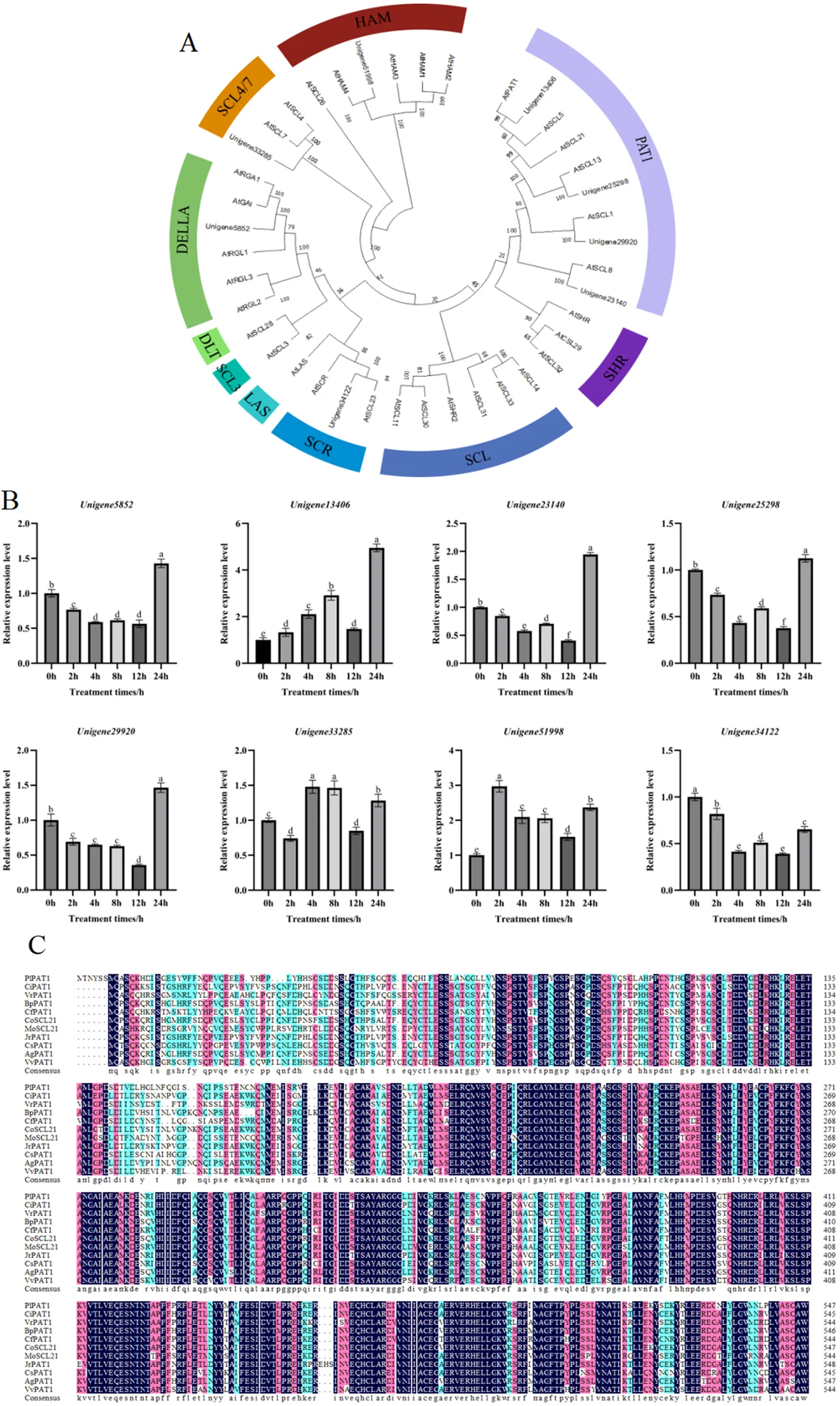
Phylogenetic trees of GRAS family members of Paeoniflora and GRAS family genes of *Arabidopsis Thaliana* were screened. Note: **A** The protein accession numbers in the Arabidopsis Information Resource (www.arabidopsis.org). **B** Expression patterns of eight P. lactiflora GRAS genes (*PlDELLA1*, *PlPAT1*, *PlSCL8*, PlSCL13, PlSCL1, PlSCL4, PlSCR1, PlHAM1) in leaves under 300 mM NaCl treatment at 0, 2, 4, 8, 12, and 24 h. **C** Amino acid sequence comparison of *PlPAT1* wih other GRAS proteins. Sequences were from *PlPAT1*: *Paeonia lactiflora*; CiPAT1: *Carya illinoinensis* (XP_042949625.1); VrPAT1: *Vitis riparia* (XP_034700766.1); BpPAT1: *Betula platyphylla* (QEE59958.1); CfPAT1: *Cornus florida* (XP_059662901.1); CoSCL21: *Corylus avellana* (XP_059441146.1); MoSCL21: *Malania oleifera* (XP_057966356.1); JrPAT1: *Juglans regia* (XP_018812730.2); CsPAT1: *Camellia sinensis* (XP_028103272.1); AgPAT1: *Alnus glutinosa* (XP_062168985.1); VvPAT1: *Vitis vinifera* (RVW50259.1)

To investigate gene expression under salt stress, qRT-PCR was performed using P. lactiflora leaves treated with salt stress at various time points. The results showed that expressions of Unigene5852, Unigene23140, Unigene25298, and Unigene29920 generally decreased and then increased after salt stress, with peak expression at 24 h. For Unigene13406, expression followed a three-phase pattern: it increased from 0 to 8 h, decreased from 8 to 12 h (but still higher than at 0 h), and then rose again from 12 to 24 h. The expression of Unigene33285 showed an initial decrease, a rise in the middle, followed by a decrease and then another increase, peaking at 4 h. Unigene34122 was down-regulated under stress, with slight increases at 8 h and 24 h, but still lower than at 0 h. Unigene51998’s expression increased early, decreased through the middle, and then rose again, with the highest expression at 2 h (Fig. 1B).

Based on domain integrity and the expression pattern under salt stress, Unigene13406, which showed relatively high and consistent up-regulation, was selected for further study. Phylogenetic analysis positioned Unigene13406 in the PAT1 branch of the GRAS family, leading to its designation as *PlPAT1*.The *PlPAT1* gene sequence is 1647 bp in length, encoding 548 amino acids. ProtParam analysis revealed a molecular weight of 61.03 kDa and a theoretical isoelectric point of 5.51. NCBI CD-search confirmed that *PlPAT1* contains a complete GRAS conserved domain at the C-terminus. Secondary structure prediction by SOPMA showed that *PlPAT1* consists of α-helix, extended strand, β-turn, and random coil, consistent with typical GRAS protein structures. Tertiary structure was predicted by SWISS-MODEL and exhibited a conserved folding pattern similar to other PAT1-type GRAS proteins. Sequence comparison with other plant species showed high similarity in the C-terminal region (Fig. 1C), and *PlPAT1* exhibited a close evolutionary relationship with AgPAT1 from Alnus glutinosa (75.81% homology).

### Analysis of *PlPAT1* expression pattern

**Fig. 2:**
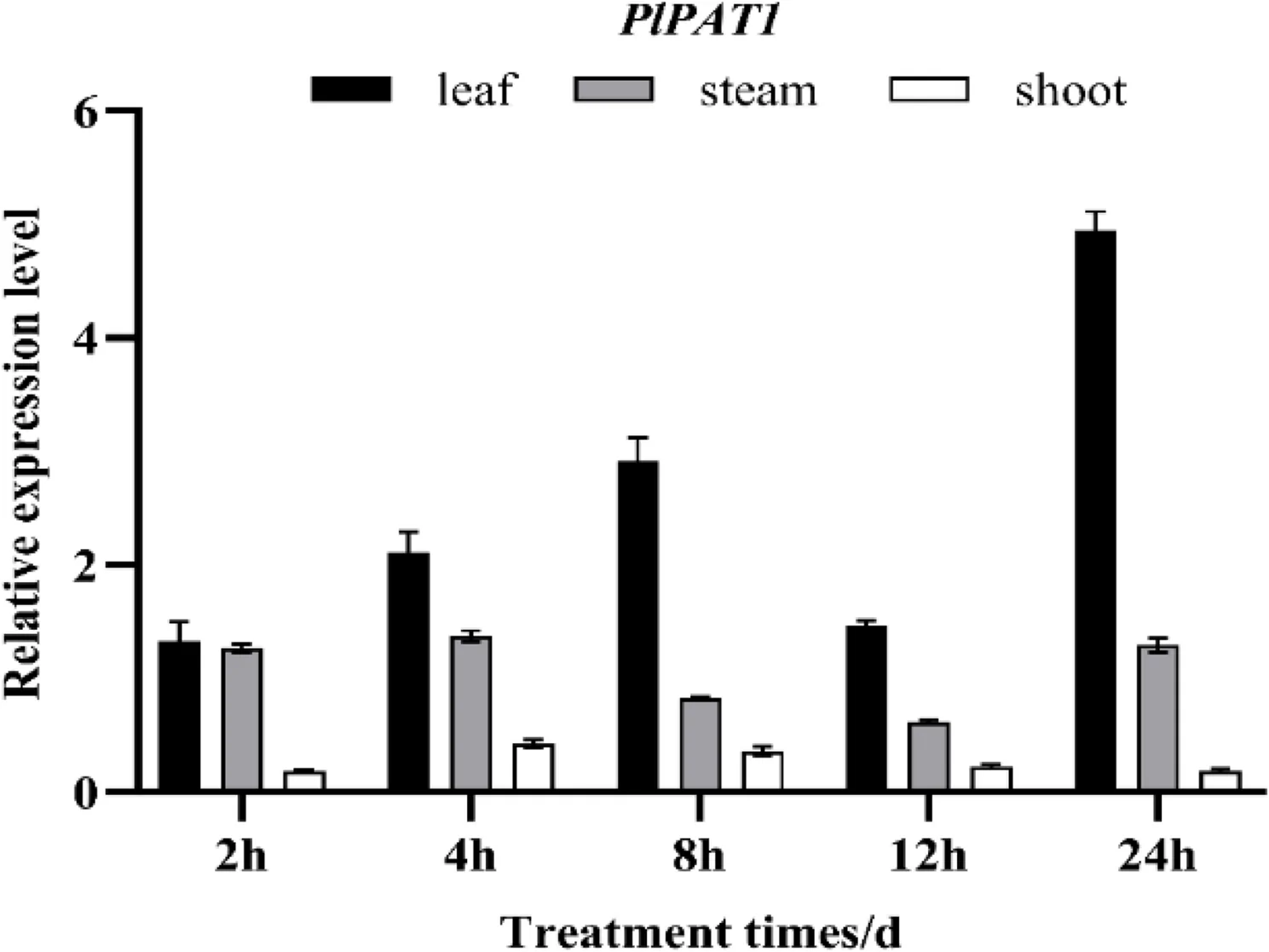
Expression patterns of *PlPAT1* in different tissues. Note: Leaves were collected at 0, 2, 4, 8, 12 and 24 h after salt stress. Actin gene as internal reference. The values are the mean ± standard error of three biological replicates. According to Duncan’s multiple range test, different letters in the column indicated significant difference (*p* < 0.05). All experiments were repeated at least three times

Under 300 mmol/L NaCl salt treatment, the relative expression levels of *PlPAT1* in leaves, stems, and roots are shown in Fig. 2. The results indicated differential expression across tissues, with up-regulated expression in leaves and stems, and down-regulated expression in roots. In leaf tissues, *PlPAT1* expression increased under salt stress, peaking at 24 h. The expression level at 12 h significantly decreased, though still higher than the untreated state. In stem tissues, *PlPAT1* expression initially increased, then decreased, and rose again, with the highest expression observed at 4 h. At 8 h and 12 h, expression was significantly lower than in the untreated state. In root tissues, PlPAT1 expression was down-regulated, with levels significantly lower than the untreated state after salt stress.

### *PlPAT1* subcellular localization results

**Fig. 3:**
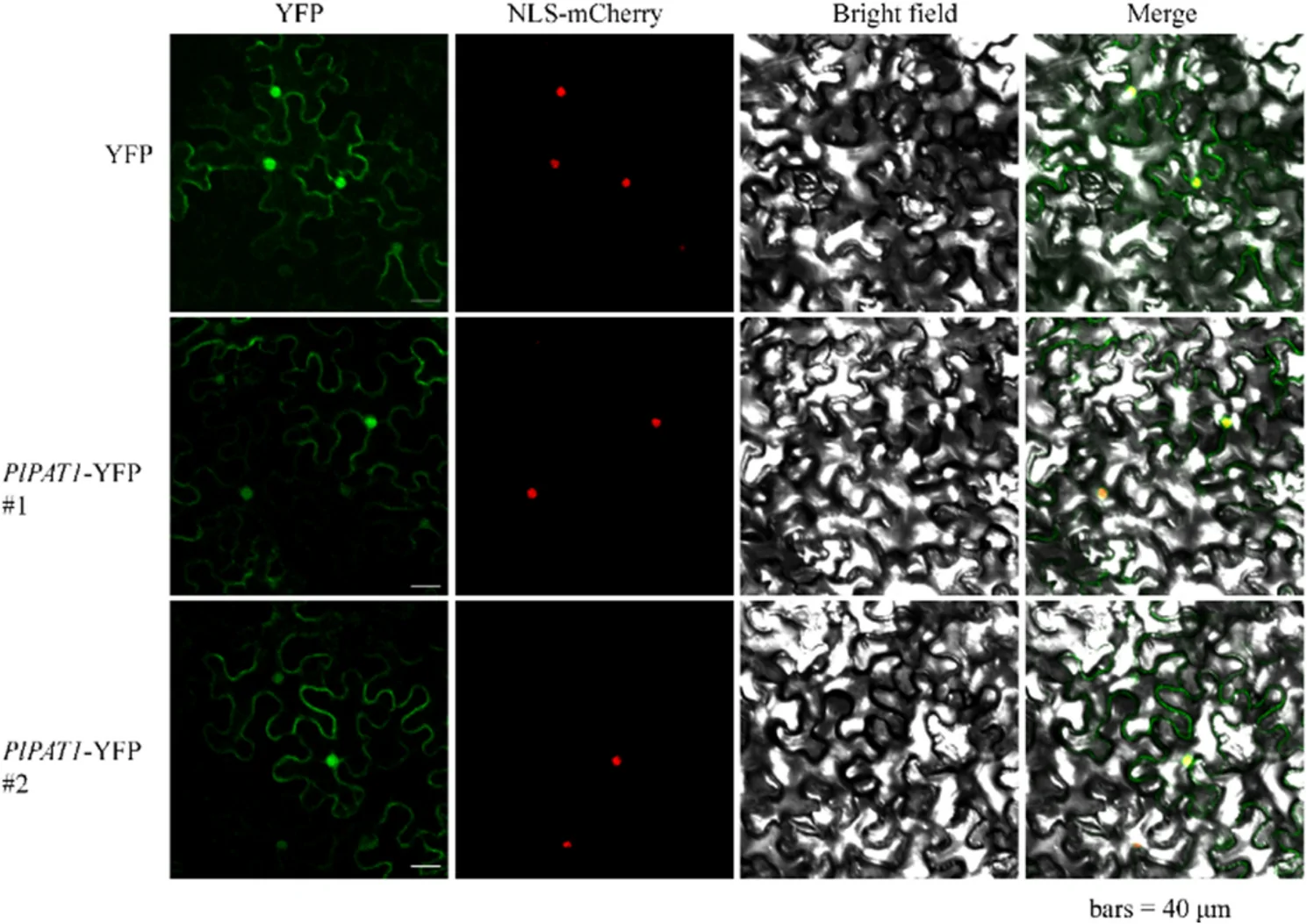
Subcellular localization of PlPAT1-YFP in tobacco epidermal cells. Scale bar = 40 μm. Note: YFP (as a control) and *PlPAT1*-YFP were briefly expressed in tobacco cells. Confocal laser scanning microscope was used to photograph YFP control group and *PlPAT1*-YFP experimental group under bright field and fluorescence field

The fusion protein was expressed in tobacco cells by injecting Agrobacterium suspensions into tobacco leaves through instantaneous transformation. The PlPAT1-YFP protein was localized using green fluorescence observed under confocal microscopy. As shown in Fig. 3, green fluorescence was detected in both the nucleus and cytoplasm of tobacco cells expressing pCambia1300S *PlPAT1*-YFP. This indicates that *PlPAT1* is localized in both the nucleus and cytoplasm, which aligns with the predictions from WOLF PSORT. The results are consistent with the localization of GmGRAS37 in soybean.

### Expression analysis of transgenic tobacco

**Fig. 4:**
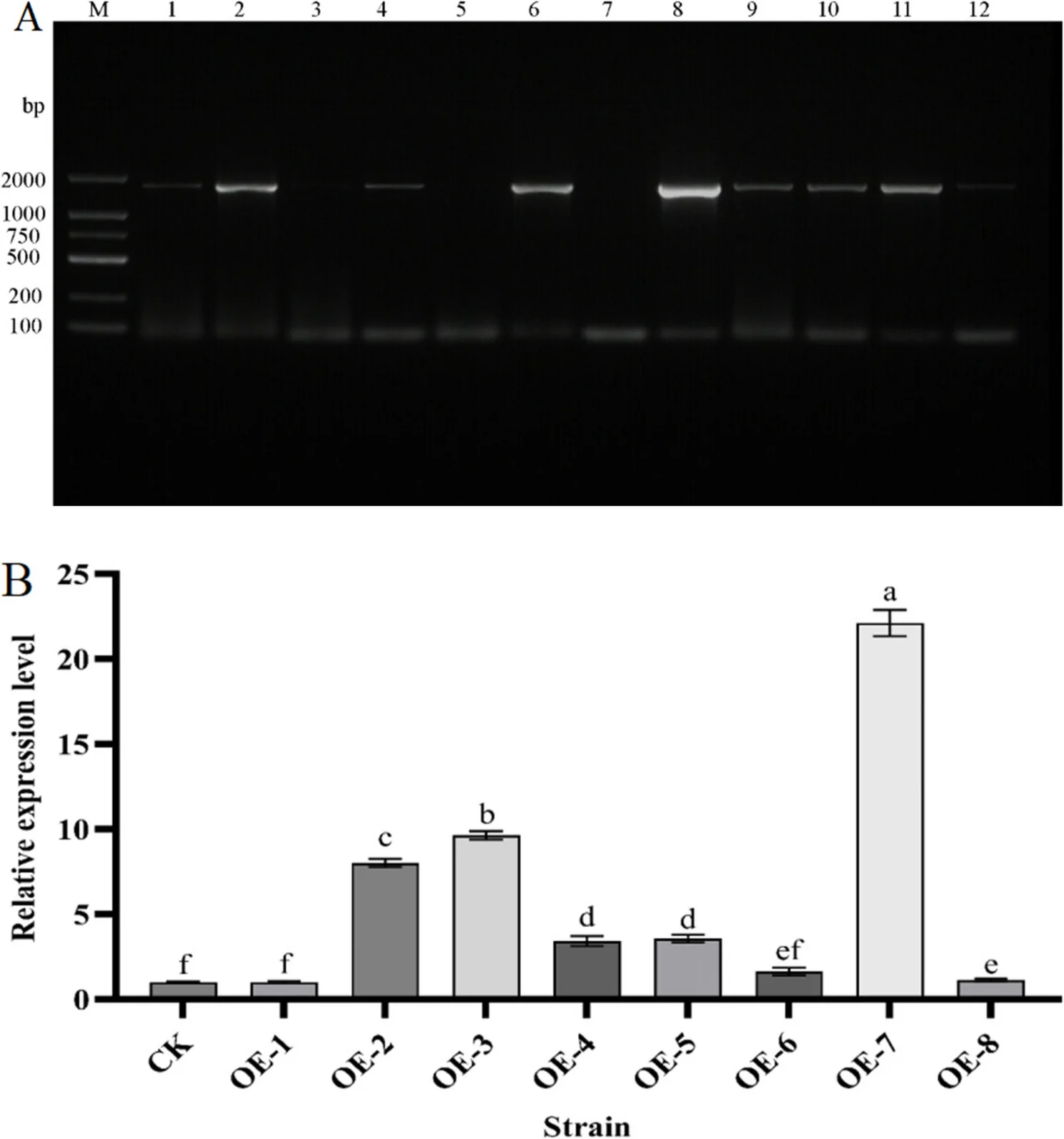
M: DNA Maker, 1 ~ 6 and 9 ~ 12: PCR products for identification materials;7:wild type tobacco PCR products, 8: recombinant plasmid PCR products. Note: **A **Expression of *PlPAT1* in different strains. Leaves of OE-1 ~ OE-8 under normal growth condition were collected, and tobacco actin gene was used as internal reference. **B** The values are the mean ± standard error of three biological replicates. According to Duncan*’*s multiple range test, different letters in the column indicated significant difference (*p* < 0.05). All experiments were repeated at least three times

An overexpression vector, PBI121-*PlPAT1*, was constructed, and transgenic tobacco plants were generated using the leaf disk method. DNA amplification of the tobacco plants confirmed the presence of the transgene, with 8 positive plants identified and renamed OE-1 to OE-8 (Fig. 4A). Leaves from the overexpressed strains were collected for RNA extraction and reverse transcription. cDNA samples were then analyzed using qRT-PCR, with tobacco actin serving as the internal reference and *PlPAT1*-RT primers for amplification.

As shown in Fig. 4B, the expression of *PlPAT1* varied across the 8 strains. OE-7 exhibited the highest relative expression, reaching 22.11, while OE-2, OE-3, OE-4, OE-5, and OE-6 showed relative expression levels ranging from 1.64 to 9.64. No significant difference in expression was observed for OE-1 and OE-8 compared to the control. Based on these results, OE-2, OE-3, and OE-7, which displayed higher expression levels, were selected for further analysis.

### Phenotypic changes of transgenic tobacco

The experimental materials included OE-2, OE-3, and OE-7 transgenic Ben’s tobacco plants overexpressing *PlPAT1*, as well as wild-type Ben’s tobacco plants at the same growth stage. Prior to treatment, the phenotypes of wild-type and overexpressed plants showed minor differences. Overexpressed plants had slightly more leaves and a more compact growth habit compared to wild-type plants.

**Fig. 5:**
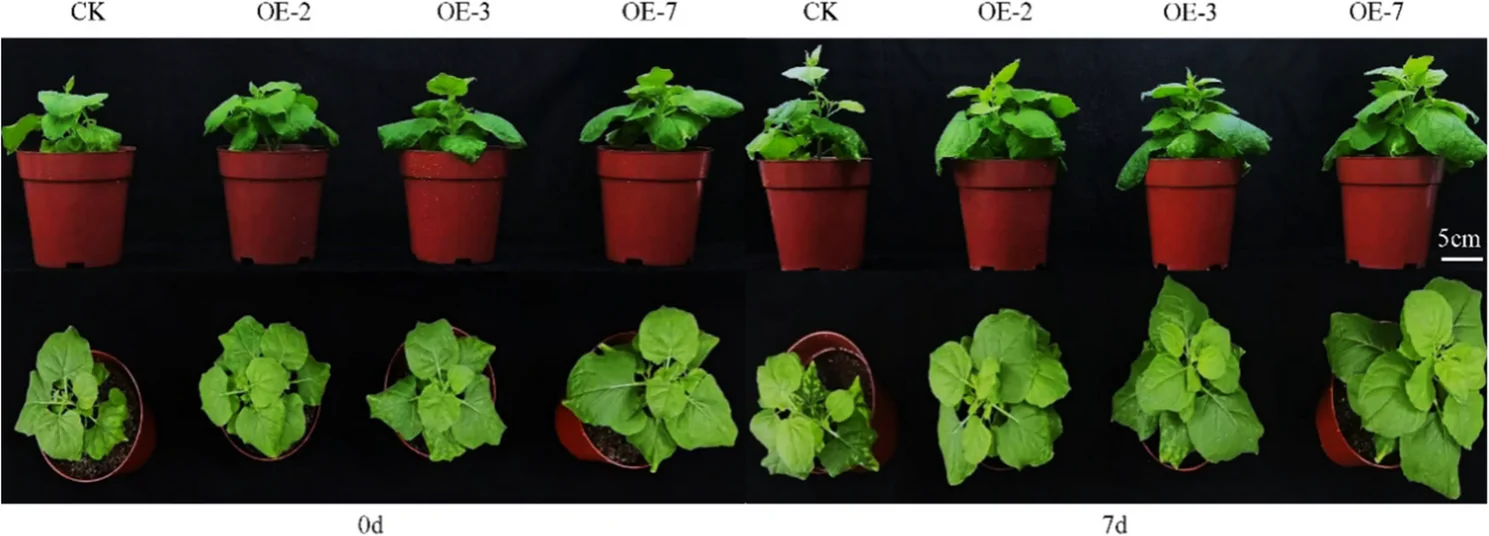
Phenotypes of wild type and *PlPAT1*-overexpressing lines (OE-2, OE-3, and OE-7) under normal and 300mmol/LNaCl salt stress for 7 days

After 7 days of salt stress treatment, morphological changes became evident in both wild-type and transgenic plants. The lower and middle leaves of both groups showed wilting and drooping, while the leaves near the roots deepened in color and exhibited increased mottling. Wild-type plants experienced more severe wilting and slower growth. In contrast, transgenic plants were less affected by the stress, with new leaves continuing to grow during the treatment period.In contrast, transgenic plants were less affected by the stress, with new leaves continuing to grow during the treatment period. Phenotypic changes were recorded and are presented in Fig. 5.

### Physiological and biochemical changes of transgenic tobacco

**Fig. 6:**
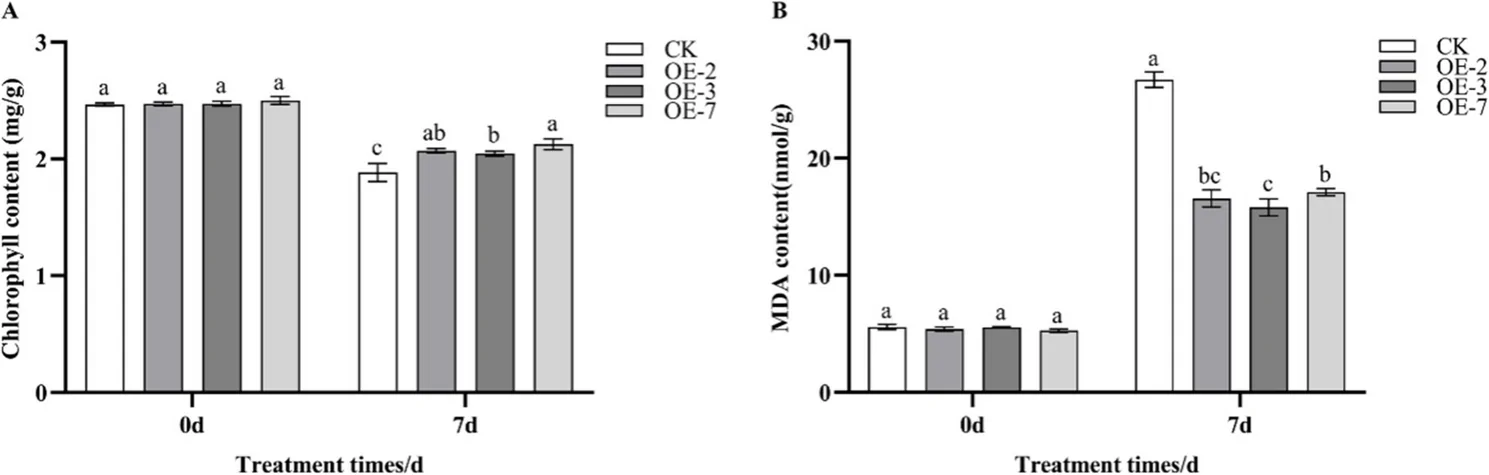
Contents of chlorophyll and malondialdehyde (MDA) in tobacco overexpressed with WT and *PlPAT1* under salt stress. Note: **A** Chlorophyll content. **B** MDA content. Three independent biological replicates were performed, Statistical significance (*p* < 0.05) is indicated by the presence of distinct lowercase letters

Under salt stress, plant ion balance is disrupted, chlorophyll synthesis is hindered, and chlorophyll stability decreases, leading to reduced chlorophyll content. Initially, the chlorophyll content was similar in both wild-type and transgenic plants. However, after salt stress treatment, transgenic plants exhibited significantly higher chlorophyll levels than wild-type plants (Fig. 6A).

Salt stress also induces an imbalance in free radical metabolism, increasing the content of superoxide free radicals, which in turn causes membrane lipid peroxidation and the production of malondialdehyde (MDA), a marker of plant stress resistance. Initially, no significant difference in MDA content was observed between wild-type and transgenic plants. After 7 days of salt stress, MDA content increased in both groups, but wild-type plants accumulated higher levels of MDA compared to transgenic plants (Fig. 6B).

**Fig. 7:**
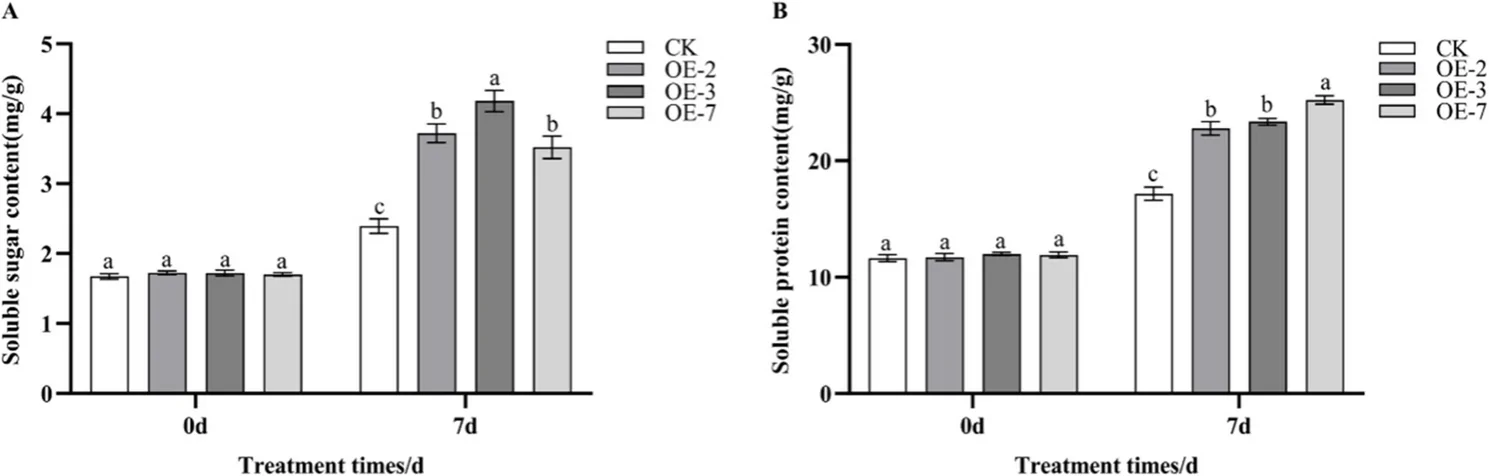
Contents of soluble sugar and soluble protein in tobacco overexpressed with WT and *PlPAT1* under salt stress. Note: **A** soluble sugar content. **B** soluble protein content. Three independent biological replicates were performed, Statistical significance (*p* < 0.05) is indicated by the presence of distinct lowercase letters

In high-salt conditions, plants adjust their ion and water balance through various physiological and biochemical processes to sustain normal growth. The accumulation of osmoregulatory substances like soluble sugars and proteins plays a key role in maintaining osmotic balance. Initially, there were no significant differences in soluble sugar and protein content between transgenic and wild-type plants. However, after salt stress treatment, transgenic plants exhibited significantly higher levels of soluble sugars (Fig. 7A) and soluble proteins (Fig. 7B) compared to wild-type plants.

The results indicate that transgenic plants experienced less damage under salt stress than wild-type plants, partly due to their higher accumulation of osmotic regulatory substances.

**Fig. 8:**
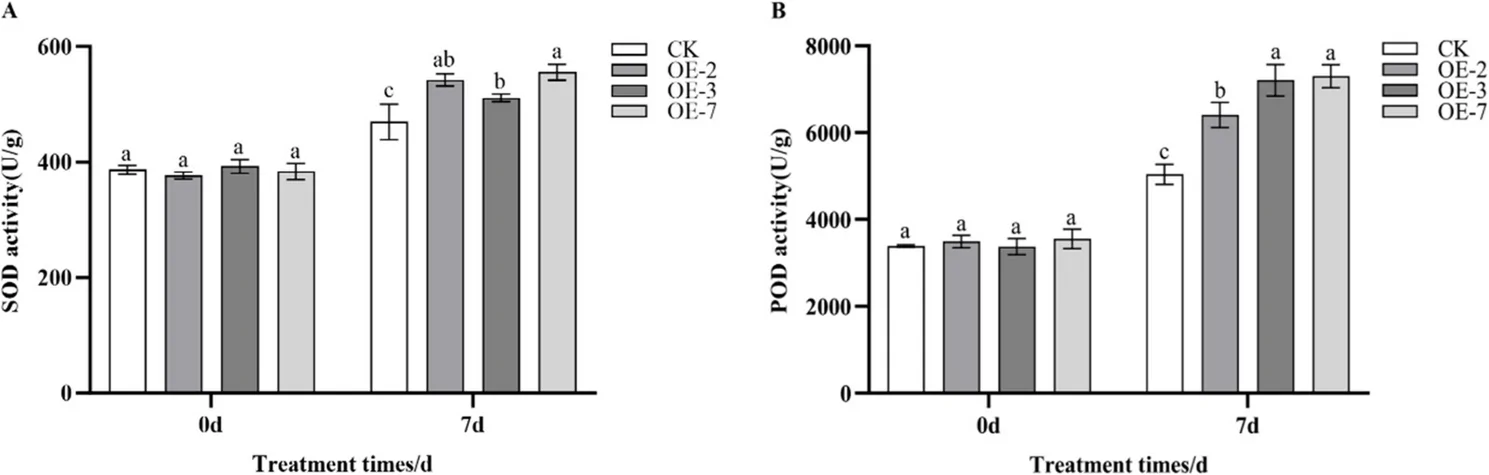
Changes of SOD and POD activities of tobacco under salt stress. Note: **A** SOD activity. **B** POD activity. Three independent biological replicates were performed, Statistical significance (*p* < 0.05) is indicated by the presence of distinct lowercase letters

Superoxide dismutase (SOD) is a key enzyme in plant defense against oxidative damage, capable of rapidly increasing its activity under stress. SOD activity in transgenic plants was significantly higher than in wild-type plants and further increased following salt stress treatment (Fig. 8A).

Peroxidase (POD) is involved in scavenging reactive oxygen species, reducing cellular peroxide accumulation. Initially, POD activity was similar in all plants. After salt stress, POD activity increased in all plants, with transgenic lines showing significantly higher activity than wild-type plants. Among transgenic lines, OE-3 and OE-7 exhibited similar POD activity, both significantly higher than OE-2 (Fig. 8B).

**Fig. 9:**
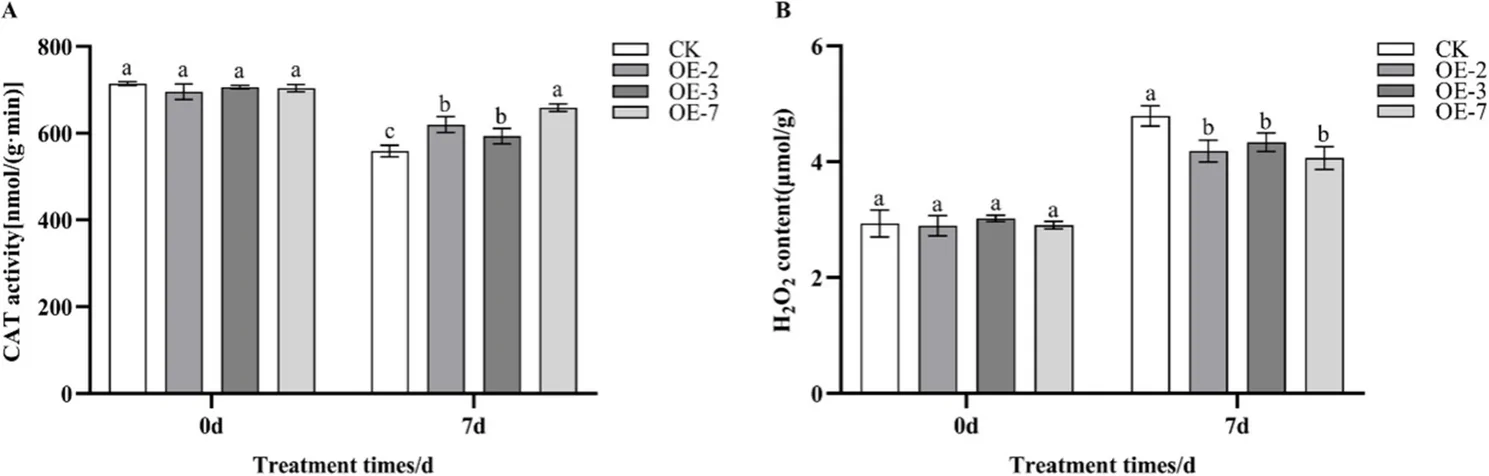
Changes of CAT activity and H_2_O_2_ content of tobacco under salt stress. Note: **A** CAT activity. **B** H_2_O_2_ content. Three independent biological replicates were performed, Statistical significance (*p* < 0.05) is indicated by the presence of distinct lowercase letters

Catalase (CAT) plays a key role in detoxifying hydrogen peroxide, alleviating oxidative damage. Initially, CAT activity was similar in all plants. After 7 days of salt stress, CAT activity decreased in all plants, but transgenic plants exhibited significantly higher CAT activity than wild-type plants (Fig. 9A).

While there was no significant difference in hydrogen peroxide content at the initial state, levels increased in all plants after salt stress. Wild-type tobacco accumulated significantly more hydrogen peroxide than transgenic plants under stress, further confirming the differences in enzyme activity between the two groups (Fig. 9B).

These results indicate that transgenic tobacco exhibited higher antioxidant enzyme activity under salt stress, effectively mitigating cell damage caused by peroxide accumulation. The hydrogen peroxide content in transgenic plants was significantly lower than in wild-type plants, further confirming the differences in enzyme activity levels between the two strains.

## Discussion

### Identification and phylogenetic analysis of GRAS gene family in *Paeonia lactiflora*

In this work, eight complete GRAS unigenes were screened from the transcriptome dataset of *P. lactiflora*. On the basis of phylogenetic clustering with *Arabidopsis* GRAS proteins, these eight sequences were grouped into four typical subfamilies, namely DELLA, PAT1, SCR and HAM [29, 30]. The classification framework adopted in this research conforms to the universal evolutionary grouping mode of angiosperm GRAS proteins summarized in multiple genome-wide identification reports of horticultural woody plants [31, 33]. Different subfamilies evolve distinct functional properties to coordinate plant growth development and abiotic stress adaptation processes. Among the four PAT1-clade genes identified here, *PlPAT1* shares 75.81% sequence similarity with *AgPAT1* from *Alnus glutinosa*, implying that the PAT1 subfamily retains high sequence conservation during woody plant evolutionary differentiation [27, 35].

Subcellular localization observation, combined with bioinformatic prediction data, showed that PlPAT1 accumulates in both the nucleus and cytoplasm and bears an intact C-terminal GRAS functional domain. The GRAS domain serves as the core structural motif that mediates the binding of transcription factors to target gene promoters [29]. Numerous published functional assays proved that most PAT1-type GRAS proteins exert transcriptional regulatory effects to modulate plant stress signaling and organ development [29, 32, 37]. Combining the structural feature and subcellular localization evidence, we infer that PlPAT1 acts as a transcriptional regulator. Further yeast one-hybrid or dual-luciferase experiments will be carried out in follow-up research to verify its transcriptional activation activity.

### Expression patterns of *PlPAT1* under salt stress

Salt treatment induced distinct tissue-specific expression changes of *PlPAT1*. The gene was significantly up-regulated in leaf and stem tissues, while its transcript abundance declined markedly in plant roots. Organ-biased expression of stress-related transcription factors is a common adaptive strategy for perennial woody species to cope with saline habitats [38]. Different plant organs undertake divergent physiological tasks under salt stress, leading to differentiated signal regulation networks.

Roots function as the primary tissue for sodium ion absorption and salt signal perception [39]. The obvious suppression of *PlPAT1* in root tissues indicates that this transcriptional regulator is negatively controlled in underground organs, which can prevent redundant signal cascade activation and guarantee the normal operation of root-specific salt resistance pathways. Similar organ-divergent expression trends have been reported in several woody PAT1 homologous genes under saline treatment, which demonstrates tissue functional differentiation of PAT1 subfamily members when plants respond to salt environments [35].

### Functional identification of *PlPAT1* in salt tolerance via heterologous expression

Heterologous overexpression of *PlPAT1* in tobacco greatly improved the salt resistance capacity of transgenic lines. Tobacco is a widely utilized heterologous expression platform for verifying gene functions of non-model woody plants, and this experimental system has been extensively applied in previous GRAS functional researches [27, 36]. After sustained NaCl treatment, transgenic tobacco maintained better growth morphology, higher chlorophyll content, and lower accumulation of MDA and H₂O₂ relative to wild-type plants. MDA and hydrogen peroxide are classic biomarkers reflecting membrane lipid peroxidation and oxidative injury under salt stress [40]. The reduced accumulation of these two substances in overexpression lines directly illustrates that PlPAT1 can alleviate salt-triggered cell membrane damage and oxidative stress [27].

### Physiological regulatory mechanism of *PlPAT1* against salt stress

Two core physiological pathways contribute to the salt tolerance mediated by PlPAT1. On the one hand, the protein activates the plant ROS scavenging system by elevating the activities of SOD, POD and CAT antioxidant enzymes. On the other hand, it promotes the accumulation of soluble sugar and soluble protein to strengthen intracellular osmotic homeostasis [14, 17]. This dual regulatory mode has been repeatedly validated in herbaceous and woody GRAS proteins responding to saline environments [27, 32, 35].

Existing studies have confirmed that many GRAS members participate in abiotic stress signal transduction through ABA and gibberellin crosstalk pathways [28, 32]. We therefore speculate that PlPAT1 may also interact with hormone signal cascades to regulate salt responses, which requires more targeted experimental evidence for confirmation. When comparing the regulatory mechanism of PlPAT1 with characterized PAT1 homologs such as *HcSCL13* and *SlGRAS40*, we found highly consistent physiological regulatory characteristics including antioxidant activation and osmotic substance accumulation, which reflects functional conservation within the PAT1 subfamily during evolution [27, 32].

The increased antioxidant enzyme activity and osmoprotectant levels in transgenic tobacco imply that PlPAT1 may modulate the transcription of downstream functional genes involved in ROS clearance and osmotic adjustment. Related regulatory modes have been validated in multiple GRAS transcription factors, which can bind to stress-responsive gene promoters to activate their expression [27, 32]. In future work, transcriptome sequencing and protein interaction screening will be conducted to excavate the direct target genes and complete regulatory cascades of PlPAT1.

### Study limitations and future perspectives

Although this study systematically characterized the sequence features, tissue expression pattern and salt-resistant function of *PlPAT1* at phenotypic and physiological levels, several limitations remain in the current research system. First, functional verification only relies on tobacco heterologous overexpression material. Due to the low efficiency of stable genetic transformation in perennial herbaceous peony, we have not constructed native *P. lactiflora* gene silencing or knockout materials to verify the endogenous gene function. Second, we only deduced that PlPAT1 serves as a transcriptional regulator according to protein localization and domain characteristics; direct transcriptional activation activity experiments have not been completed in this manuscript. Third, the upstream regulatory factors of *PlPAT1* and the complete hormone-related signal network linked to this gene under salt stress are still unclear. To fill these gaps, we plan to carry out yeast two-hybrid screening, transient transformation dual-luciferase detection and peony genetic transformation trials in follow-up research, so as to fully resolve the molecular regulatory network of PlPAT1 under salt stress.

## Conclusion

In summary, this study identified eight GRAS family genes from *Paeonia lactiflora* and functionally characterized a PAT1-type transcription factor gene *PlPAT1*. *PlPAT1* showed tissue-specific expression under salt stress, with upregulation in leaves and stems and downregulation in roots. The PlPAT1 protein was localized in the nucleus and cytoplasm and exhibited typical characteristics of a transcriptional regulator. Heterologous overexpression of *PlPAT1* in tobacco significantly enhanced salt tolerance by improving reactive oxygen species scavenging and osmotic adjustment ability. These results indicate that PlPAT1 acts as a positive regulator in plant salt stress response and provides a valuable gene resource for molecular breeding of stress-resistant plants.

## Supplementary Information

Supplementary Material 1.

Supplementary Material 2.

Supplementary Material 3.

## Data Availability

The nucleotide sequence of the PlPAT1 gene from Paeonia lactiflora reported in this paper has been deposited in the NCBI GenBank database under the accession number PV053163. The associated protein sequence can be accessed through the same record. All other data generated or analyzed during this study are included in this published article and its supplementary information files.

## Abbreviations

SOD: superoxide dismutase
POD: peroxidase
CAT: catalase
MDA: malondialdehyde
ROS: reactive oxygen species
NaCl: sodium chloride
qRT-PCR: quantitative real-time polymerase chain reaction
YFP: yellow fluorescent protein
WT: wild type
OE: overexpression line
ChIP-seq: chromatin immunoprecipitation sequencing

## References

[1] Zhu JK. Abiotic Stress Signaling and Responses in Plants. Cell. 2016;167(2):313–24. 10.1016/j.cell.2016.08.029 PMC5104190 27716505

[2] Morton MJL, Awlia M, Al-Tamimi N, Saade S, Pailles Y, Negrão S, Tester M. Salt stress under the scalpel - dissecting the genetics of salt tolerance. Plant J. 2019;97(1):148–63. 10.1111/tpj.14189 PMC6850516 30548719

[3] Balasubramaniam T, Shen G, Esmaeili N, Zhang H. Plants’ Response Mechanisms to Salinity Stress. Plants (Basel). 2023;12:12. 10.3390/plants12122253 PMC10300796 37375879

[4] Gong Z, Xiong L, Shi H, Yang S, Herrera-Estrella LR, Xu G, Chao DY, Li J, Wang PY, Qin F, Li J, Ding Y, Shi Y, Wang Y, Yang Y, Guo Y, Zhu JK. Plant abiotic stress response and nutrient use efficiency. Sci China Life Sci. 2020;63(5):635–74. 10.1007/s11427-020-1683-x 32246404

[5] Negrão S, Schmöckel SM, Tester M. Evaluating physiological responses of plants to salinity stress. Ann Bot. 2017;119(1):1–11. 10.1093/aob/mcw191 PMC5218372 27707746

[6] Yadav SP, Bharadwaj R, Nayak H, Mahto R, Singh RK, Prasad SK. Impact of salt stress on growth, productivity and physicochemical properties of plants: A Review. Int J Chem Stud. 2019;7(2):1793–8.

[7] Munns R. Genes and salt tolerance: bringing them together. New Phytol. 2005;167(3):645–63. 10.1111/j.1469-8137.2005.01487.x 16101905

[8] Abbasi H, Jamil M, Haq A, Ali S, Ahmad R, Malik Z, Parveen. Salt stress manifestation on plants, mechanism of salt tolerance and potassium role in alleviating it: a review. Zemdirbyste-Agriculture. 2016;103(2):229–38.

[9] Sdouga D, Ben Amor F, Ghribi S, Kabtni S, Tebini M, Branca F, Trifi-Farah N, Marghali S. An insight from tolerance to salinity stress in halophyte Portulaca oleracea L.: Physio-morphological, biochemical and molecular responses. Ecotoxicol Environ Saf. 2019;172:45–52. 10.1016/j.ecoenv.2018.12.082 30677744

[10] Sun Z, Qi X, Wang Z, Li P, Wu C, Zhang H, Zhao Y. Overexpression of TsGOLS2, a galactinol synthase, in *Arabidopsis thaliana* enhances tolerance to high salinity and osmotic stresses. Plant Physiol Biochem. 2013;69:82–9. 10.1016/j.plaphy.2013.04.009 23728391

[11] Mansour MMF, Ali EF. Evaluation of proline functions in saline conditions. Phytochemistry. 2017;140:52–68. 10.1016/j.phytochem.2017.04.016 28458142

[12] Rehman AU, Bashir F, Ayaydin F, Kóta Z, Páli T, Vass I. Proline is a quencher of singlet oxygen and superoxide both in in vitro systems and isolated thylakoids. Physiol Plant. 2021;172(1):7–18. 10.1111/ppl.13265 33161571

[13] Wei JT, Zhao SP, Zhang HY, Jin LG, Yu TF, Zheng L, Ma J, Chen J, Zhou YB, Chen M, Fu JD, Ma YZ, Xu ZS. GmDof41 regulated by the DREB1-type protein improves drought and salt tolerance by regulating the DREB2-type protein in soybean. Int J Biol Macromol. 2023;230:123255. 10.1016/j.ijbiomac.2023.123255 36639088

[14] Singh P, Choudhary KK, Chaudhary N, Gupta S, Sahu M, Tejaswini B, Sarkar S. Salt stress resilience in plants mediated through osmolyte accumulation and its crosstalk mechanism with phytohormones. Front Plant Sci. 2022;13:1006617. 10.3389/fpls.2022.1006617 PMC9552866 36237504

[15] Yin YG, Kobayashi Y, Sanuki A, Kondo S, Fukuda N, Ezura H, Sugaya S, Matsukura C. Salinity induces carbohydrate accumulation and sugar-regulated starch biosynthetic genes in tomato (Solanum lycopersicum L. cv. ‘Micro-Tom’) fruits in an ABA- and osmotic stress-independent manner. J Exp Bot. 2010;61(2):563–74. 10.1093/jxb/erp333 PMC2803223 19995825

[16] Eggert E, Obata T, Gerstenberger A, Gier K, Brandt T, Fernie AR, Schulze W, Kühn C. A sucrose transporter-interacting protein disulphide isomerase affects redox homeostasis and links sucrose partitioning with abiotic stress tolerance. Plant Cell Environ. 2016;39(6):1366–80. 10.1111/pce.12694 26670204

[17] Li Z, Zhu L, Zhao F, Li J, Zhang X, Kong X, Wu H, Zhang Z. Plant Salinity Stress Response and Nano-Enabled Plant Salt Tolerance. Front Plant Sci. 2022;13:843994. 10.3389/fpls.2022.843994 PMC8981240 35392516

[18] Hou Q, Ufer G, Bartels D. Lipid signalling in plant responses to abiotic stress. Plant Cell Environ. 2016;39(5):1029–48. 10.1111/pce.12666 26510494

[19] Azeem M, Pirjan K, Qasim M, Mahmood A, Javed T, Muhammad H, Yang S, Dong R, Ali B, Rahimi M. Salinity stress improves antioxidant potential by modulating physio-biochemical responses in Moringa oleifera Lam. Sci Rep. 2023;13(1):2895. 10.1038/s41598-023-29954-6 PMC9938910 36807545

[20] Wang M, Yuan J, Qin L, Shi W, Xia G, Liu S. TaCYP81D5, one member in a wheat cytochrome P450 gene cluster, confers salinity tolerance via reactive oxygen species scavenging. Plant Biotechnol J. 2020;18(3):791–804. 10.1111/pbi.13247 PMC7004906 31472082

[21] Chen TC, Chou SY, Chen MC, Lin JS. IbTLD modulates reactive oxygen species scavenging and DNA protection to confer salinity stress tolerance in tobacco. Plant Sci. 2022;323:111415. 10.1016/j.plantsci.2022.111415 35963494

[22] Zhou J, Qiao J, Wang J, Quan R, Huang R, Qin H. OsQHB Improves Salt Tolerance by Scavenging Reactive Oxygen Species in Rice. Front Plant Sci. 2022;13:848891. 10.3389/fpls.2022.848891 PMC9115556 35599895

[23] Lu C, Liu X, Tang Y, Fu Y, Zhang J, Yang L, Li P, Zhu Z, Dong P. A comprehensive review of TGA transcription factors in plant growth, stress responses, and beyond. Int J Biol Macromol. 2024;258(Pt 1):128880. 10.1016/j.ijbiomac.2023.128880 38141713

[24] Saibo NJ, Lourenço T, Oliveira MM. Transcription factors and regulation of photosynthetic and related metabolism under environmental stresses. Ann Bot. 2009;103(4):609–23. 10.1093/aob/mcn227 PMC2707349 19010801

[25] Khan SA, Li MZ, Wang SM, Yin HJ. Revisiting the Role of Plant Transcription Factors in the Battle against Abiotic Stress. Int J Mol Sci. 2018;19:6. 10.3390/ijms19061634 PMC6032162 29857524

[26] Jin J, Tian F, Yang DC, Meng YQ, Kong L, Luo J, Gao G. PlantTFDB 4.0: toward a central hub for transcription factors and regulatory interactions in plants. Nucleic Acids Res. 2017;45(D1):D1040–5. 10.1093/nar/gkw982 PMC5210657 27924042

[27] Zhang S, Li X, Fan S, Zhou L, Wang Y. Overexpression of HcSCL13, a Halostachys caspica GRAS transcription factor, enhances plant growth and salt stress tolerance in transgenic Arabidopsis. Plant Physiol Biochem. 2020;151:243–54. 10.1016/j.plaphy.2020.03.020 32240936

[28] Ito T, Fukazawa J. SCARECROW-LIKE3 regulates the transcription of gibberellin-related genes by acting as a transcriptional co-repressor of GAI-ASSOCIATED FACTOR1. Plant Mol Biol. 2021;105(4–5):463–82. 10.1007/s11103-020-01101-z 33474657

[29] Lee MH, Kim B, Song SK, Heo JO, Yu NI, Lee SA, Kim M, Kim DG, Sohn SO, Lim CE, Chang KS, Lee MM, Lim J. Large-scale analysis of the GRAS gene family in *Arabidopsis thaliana*. Plant Mol Biol. 2008;67(6):659–70. 10.1007/s11103-008-9345-1 18500650

[30] Zhang B, Liu J, Yang ZE, Chen EY, Zhang CJ, Zhang XY, Li FG. Genome-wide analysis of GRAS transcription factor gene family in Gossypium hirsutum L. BMC Genomics. 2018;19(1):348. 10.1186/s12864-018-4722-x PMC5944045 29743013

[31] Lu X, Liu W, Xiang C, Li X, Wang Q, Wang T, Liu Z, Zhang J, Gao L, Zhang W. Genome-Wide Characterization of GRAS Family and Their Potential Roles in Cold Tolerance of Cucumber (Cucumis sativus L). Int J Mol Sci. 2020;21:11. 10.3390/ijms21113857 PMC7312588 32485801

[32] Liu Y, Huang W, Xian Z, Hu N, Lin D, Ren H, Chen J, Su D, Li Z. Overexpression of SlGRAS40 in Tomato Enhances Tolerance to Abiotic Stresses and Influences Auxin and Gibberellin Signaling. Front Plant Sci. 2017;8:1659. 10.3389/fpls.2017.01659 PMC5622987 29018467

[33] Kumari P, Gahlaut V, Kaur E, Singh S, Kumar S, Jaiswal V. Genome-Wide Identification of GRAS Transcription Factors and Their Potential Roles in Growth and Development of Rose (< i>Rosa chinensis). J Plant Growth Regul. 2023;42(3):1505–21.

[34] Park HJ, Jung WY, Lee SS, Song JH, Kwon SY, Kim H, Kim C, Ahn JC, Cho HS. Use of heat stress responsive gene expression levels for early selection of heat tolerant cabbage (Brassica oleracea L). Int J Mol Sci. 2013;14(6):11871–94. 10.3390/ijms140611871 PMC3709761 23736694

[35] He Z, Tian Z, Zhang Q, Wang Z, Huang R, Xu X, Wang Y, Ji X. Genome-wide identification, expression and salt stress tolerance analysis of the GRAS transcription factor family in Betula platyphylla. Front Plant Sci. 2022;13:1022076. 10.3389/fpls.2022.1022076 PMC9638169 36352865

[36] Ni L, Wang Z, Liu X, Wu S, Hua J, Liu L, Yin Y, Li H, Gu C. Genome-wide study of the GRAS gene family in Hibiscus hamabo Sieb. et Zucc and analysis of HhGRAS14-induced drought and salt stress tolerance in Arabidopsis. Plant Sci. 2022;319:111260. 10.1016/j.plantsci.2022.111260 35487668

[37] Torres-Galea P, Hirtreiter B, Bolle C, Two GRAS. proteins, SCARECROW-LIKE21 and PHYTOCHROME A SIGNAL TRANSDUCTION1, function cooperatively in phytochrome A signal transduction. Plant Physiol. 2013;161(1):291–304. 10.1104/pp.112.206607 PMC3532260 23109688

[38] Hao XL, Gong YY, Chen SX, Ma CQ, Duanmu HZ. Genome-Wide Identification of GRAS Transcription Factors and Their Functional Analysis in Salt Stress Response in Sugar Beet. Int J Mol Sci. 2024;25(13):7132–7132. 10.3390/ijms25137132 PMC11241673 39000240

[39] Bisht A, Eekhout T, Canher B, Lu R, Vercauteren I, De Jaeger G, Heyman J, De Veylder L. PAT1-type GRAS-domain proteins control regeneration by activating DOF3.4 to drive cell proliferation in Arabidopsis roots. Plant Cell. 2023;35(5):1513–31. 10.1093/plcell/koad028 PMC10118276 36747478

[40] Farmer EE, Mueller JM. Lipid Peroxidation and RES-Activated Signaling. Annu Rev Plant Biol. 2013;64(1):429–50. 10.1146/annurev-arplant-050312-120132 23451784

