# Supplementary material for: Overexpression of PlPAT1, a GRAS transcription factor from Paeonia lactiflora, enhances salt stress tolerance in transgenic tobacco
Source: BMC Genomics. 2026 Aug 1;27:892. doi: 10.1186/s12864-026-13204-w (PMC13718014; doi:10.1186/s12864-026-13204-w)
Supplement: Supplementary file 1 [file 12864_2026_13204_MOESM2_ESM.pdf]

Supplementary Note 1: Experimental Documentation for Figure 4A

1. Statement of Data Availability

The original, uncropped digital image for the PCR verification gel (Figure 4A) is unavailable due to a local storage device failure. The following documentation is provided to affirm the authenticity and validity of the experiment.

2. Experimental Protocol Summary

PCR Amplification: The reaction was performed using with specific primers for the PlPAT1 transgene and the tobacco actin reference gene.

Gel Electrophoresis: PCR products were separated on a 1.0% agarose gel stained with GelRed at 120V for 30 minutes.

3. Gel Lane Identification

The following sample loading order corresponds to the cropped image in Figure 4A of the manuscript:**Lane M:** DNA Molecular Weight Marker

Lanes 1-6, 9-12: Independent transgenic tobacco lines (OE-1 to OE-8)

Lane 7: Wild-type (WT) tobacco control

Lane 8: Plasmid (pBI121-PlPAT1) positive control

4. Supporting Raw Data

Attached below are scanned pages from the original laboratory notebook recorded at the time of the experiment. These pages detail the sample preparation, PCR setup, and gel analysis for the results presented in Figure 4A.

RNA提取

诺唯赞试剂盒 ①称量植物叶片 0.2g+，分两管，每管半管以上粉末
②刺槐 PSL 裂解液，烟草 EL
③研磨后用漂浮板将管浸入液氮中，不可进入管内
④ 40ul/管洗脱 (RNase Free H_2O)，+1管量提前预热
⑤再洗脱一次，增大浓度
⑥分装 RNA 测，跑胶，反转录，预备第3管以上
⑦尽量灭无酶枪头
⑧计数 蓝枪头 每管 7个，黄枪头每管2个，外加吸沉淀体2个
白枪头分装每管 1个起步，检测耗最多，建议装满

反转录

一、用普通 PCR
华越洋
20ul { RNA 500ng
oligo(dT) 1ul 42℃ 50min，85℃ 5sec
5X Mix 4ul 若qPCR ↓ 15min
gDNA Remover 1ul
RNase free H_2O to 20

①试剂使用前轻混
②加完后轻轻吹打，不可旋涡

二、用于 qPCR
1步 16ul { RNase-free ddH_2O to 16 轻吹混
4x Mix 4ul 42℃ 2min
RNA 500ng
2步 { 1步反应液 16ul 轻吹混
5X Mix 4ul 50℃ 15min，85℃ 5sec

试剂盒使用前，4x，5x 试剂短暂离心，轻吹混，全程不可过度旋涡振荡。

引物设计

普通PCR引物

①引物长度 18–30nt，最好 18–25，上下游引物长度不太于3.

②引物3'端最后一个碱基最好为 G/C，避免：高GC/AT含量区域(3个以上)

③正反向引物 Tm 相差不超过1℃为佳，使用 premier 5 计算 Tm 为 55–65℃

↓ 实为 50–60℃

④引物 GC 含量 40%–60%之间 4种碱基均匀分配

⑤引物内部或两条引物之间避免 5个碱基以上的互补序列.

⑥尽可能跨内含子设计引物，限制 gDNA的扩增.

⑦使用BLAST检索引物特异性，避免非特异性扩增
（物种基因组数据库搜索也可）

2. 定量PCR引物

补充：

①引物18–25nt，产物长度 80–150bp 最佳

②引物 Tm 调整接近 60℃

qPCR：

Vazyme 2x AceQ Universal SYBR qPCR Master Mix（诺唯赞）

0h	2h	4h	8h
0 0 0	0 0 0	000	000

1、罗氏白色八联管 可放 12列，与盖子可不灭菌 八联管照紫外即可

2、每个基因在某一时段设置三个生物学重复，每个时间段 同一板上必须设置相应内参

3、提前计算 预混液量，Mix、水、cDNA 提前按+2孔量 预混
引物 0.8 ul 单独加

4、初次定量级 设置不同稀释倍数如 x5，x10 x20 x30 cDNA稀释

5、Mix及剩预混液全程避光，冰上保存，短暂离心、吹打预混液
实验应于超净工作台避光进行。八联管先磕桌面，使侧壁液体流下去，然后离心（两两对称，加每三列拿去离心，在旧PCR仪加盖）
6、刷卡开机 → 开PCR仪开关 → 开软件，连接成功 → 打开温度模板 →
Next，Create New → 全选（看侧的范围），SYBR：全选，unknown → 竖选（3列）
0h，横选，A基因 → 全选，生物学重复3，show，显示无误，保存模板（可创建文件夹）→ 打开（open），放置八联管，勿触碰旁边及盖子，黑色板按压持平 → close，开始，保存数据至...，结束后再选 excel...

PCR

① 普通PCR 按加样顺序（生工） 冰上或冰盒加样

25ul 体系

水	9.5	预变性	94℃	5min	
引物	F+R: 1+1	变性	94℃	30sec	35
模板	1 (500ng/ul)	退火	50-60℃	30sec	
Mix	12.5	延伸	72	30-60sec/kb	
		终延伸	72	10min	
		保存	4℃	∞	

② 高保真酶

TIANGEN 50ul

cDNA	0.2ul (500ng/ul)	98℃	30sec	
F+R	5ul	98℃	10sec	×35
Mix	25ul	60℃	20sec	
ddH_2O	19.8	72℃	30sec/kb	
		72℃	5min	
		4℃	∞	

YE SEN：50ul

ddH_2O	19.2ul	预变性	98℃	3min	
cDNA	0.8 (500ng/ul)	变性	98℃	10sec	×35
F+R	5ul	退火	60℃	20sec	
Mix	25ul	延伸	72℃	30sec/kb	
		终延伸	72℃	5min	
		保存	4℃	∞	

Vazyme：50ul

ddH_2O	20.2ul	预变性	95℃	3min	
Mix	25ul	变性	95℃	15sec	×35
F+R	4ul	退火	60℃	15sec	
cDNA	0.8ul (500ng/ul)	延伸	72℃	60sec/kb	
		终延伸	72℃	5min	

胶回收（生工）

① 高保真酶的PCR，跑胶新鲜电泳液，0.8%的胶（0.24/30ml）
② 宰实验服，戴两层手套，解剖刀酒精消毒、
③ 1.5ml 离心管 切胶前后称重，标记，记数值
④ 胶有厚度，切胶放倒，只保留条带部分，多余应切除、
尽量胶块质量 100mg ≤ M ≤ 400mg.
⑤ 50°C 金属浴 溶解，中间每隔1min 间或颠倒混匀 胶块溶化拿出
⑥ 对于浓度不足的胶，可选择同一基因四个管
第一管 用 45ml H_2O 正常洗脱两次，洗脱液再用于第2管洗脱两次
每次加洗脱液静置 1min，可提高浓度 最终得两管

连A尾，按比例加大体系 （TIANGEN）

如1.8倍
20 → 36ul

DNA	27ml	
Buffer	7.2ml	轻混 72°C，30min
Taq	1.8ml	（PCR仪）

A尾结束，胶回收 用于连接反应

连接

T载 → PMD19－T vector（高效克隆PCR产物的专用载体）
2692 bp 抗性 Ampr

克隆，Vector DNA 与 Insert DNA mol 比：1:2～1:10
一般选取 1:2、1:3

插入片段应加体积 ＝ T载体量 × 目的基因长度 ÷ T载长度 × $\frac{\text{目的基因}}{\text{T载}}$ ÷ 插入片段浓度
50ng　1.17kbp　2.692kbp　2 or 3　ng/ml
1.095kbp

紫外照射

T载连接 ①DNA aq 5ml体系

		1:2	1:3
	T-vector	1ml	1ml
	DNA	4ml	
	dd H_2O		

②加入 5ml DNA Ligation kit

③16°C 反应 30min (PCR 仪)

④10ml 加入 100ml DH5α 感受态中，冰上放置 30min

提前 8min 解冻，枪头轻搅 / 手轻敲管壁

轻轻插进冰里

⑤42°C 水浴 45s，冰上 1min

⑥加入 890ml 液体 LB (无Amp) 37°C 150rpm 60min 振荡（放入 50ml 离心管）

4000 rpm，5min 离心

⑦吸 800ml 上清弃置，剩 200ml 吹打混匀，取 100ml/50ml 混合液涂布在含 X-Gal、IPTG、Amp 的 LB 固体培养基上（4板/100ml）

（生工）→ 100ml/100ml 2板 1:2，2板 1:3

⑧菌液吸干后，倒置 37°C 过夜培养（12-16h），4°C 冰箱 3天起步

⑨含 Amp 的 LB 液体培养基分 12 管于 15ml 离心管中，每管 5ml

大枪头套小枪头，挑取单一白色菌落，37°C，200rpm 12h-16h，

摇晃，使沉淀溶于菌液，

于超净工作台内加样，进行菌检 PCR

13管	65	mix
	45.5	H_2O
	13	F+R

10ml 体系 ×(12+1)管

⑩有明亮条带保留，送检 6管（1ml/管），

先 200ml 30% 甘油，200ml 菌液，振荡混匀，-80°C（保存菌液）

公司构完载体→菌液、质粒(不用)

第一天
① 甘油菌解冻，分出 200ml 至 2/1.5ml 离心管中
② 用接种环蘸取菌液 在 LB 固体培养基上 平板划线 (含 kana)

二天
③ 37℃ 培养 12h-16h，(二区) 二区长出菌落 放入4℃冰箱
④ 4℃ 当天晚上 挑单克隆，37℃，200rpm 12h (倒6ml 菌液)

三天
⑤ 菌检，挑出明亮条带2管，200ml 甘 + 200 菌液 冻管保存
⑥ 剩余菌液用于 提质粒

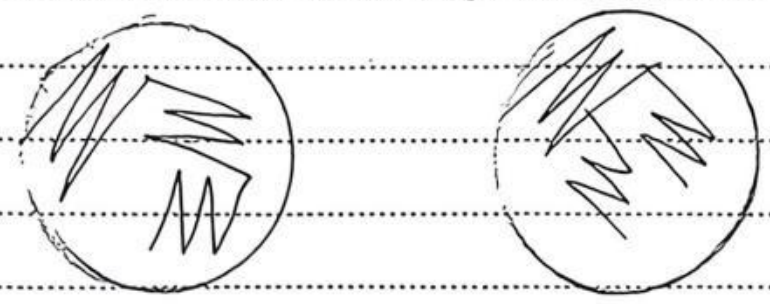

质粒小提 (天根)

① 新鲜过夜培养菌液 (6ml - 600ml (保菌) 7 5ml)

② 5ml 每次取1ml 离心，尽量吸取上清，收集沉淀于同一离心管

③ 加入 RNase A 酶的 P1 4℃冰箱 保存 6个月

④ 提取结束，检测浓度，比值

重组质粒转化农杆菌（冻融法）

1、-80℃取出GV3101感受态细胞，缓缓插入冰中，融化（1h+）

2、1-10ml重组质粒（200-1000ng）加入100ml感受态细胞，枪头轻搅混，封口
↑设置2-3个浓度梯度

3、冰上5min，液氮5min，37℃水浴5min，冰浴5min

4、加入900ml YEB液体培养基，混匀颠倒于50ml离心管中28℃，100rpm
振荡3h。

5、4000rpm，5min室温下离心，除800ml上清，剩200ml吹打混匀。

6、吸取100ml/150ml于YEB固体培养基平板上，涂布，封口，28℃倒置
培养2-3d。
（YEB：100ml Kana + 100ml Rif /100ml；50mg/mL）

7、挑单克隆于5ml YEB液体培养基中（15ml离心管），28℃，200rpm，
培养2d（或3d）

8、菌液PCR，甘油菌种保藏，每管x（4+）分装，-80℃保存

农杆菌复苏

① 甘油菌平板划线（4板） YEB 1:100ul kan + 100ul Rif）
倒置培养3d，28℃

② 挑单克隆（12管） 28℃，200rpm 2d（小摇）

③ PCR菌检，选取4管+ （白天）

④ 大摇（晚上起）：(1) 每管取4ml菌液分为2ml离心管×2管，
4000rpm，5min离心，去上清，剩余1ml菌液分别吹打
即1管吹打完吸液体于第2管中吹打，使菌体重悬。

(2) 重悬菌液置于50ml YEB液体培养基（50ul kan+Rif）混匀，每瓶
吸取1-2ml保存于离心管中作为调零管（4℃保存），剩余混合
液 28℃，200rpm培养

上午

⑤ 培养8h后，开始测值：[illegible]，未摇的菌液调零管不可扔，
直至 OD_{600}：0.6~0.8。

⑥ 选取其中2瓶菌液转移至50ml离心管，密封，
4000rpm，4℃，20min集菌，弃上清（弃前看体积）
↓前一天配制，烘箱放置（已灭菌）

⑦ 用等体积MS液体培养基重悬保菌体，加入AS（100umol/L），
黑暗条件放置1h复苏，复苏后作侵染液，转化烟草
