# Supplementary material for: Overexpression of PlPAT1, a GRAS transcription factor from Paeonia lactiflora, enhances salt stress tolerance in transgenic tobacco
Source: BMC Genomics. 2026 Aug 1;27:892. doi: 10.1186/s12864-026-13204-w (PMC13718014; doi:10.1186/s12864-026-13204-w)
Supplement: Supplementary file 2 [file 12864_2026_13204_MOESM1_ESM.docx]

Additional file 1: List of PCR primers used in this study

| No. | Name | Oligonucleotides (5*'*-3*'*) | Note |
| --- | --- | --- | --- |
| P1  P2  P3  P4  P5  P6  P7  P8  P9  P10  P11  P12  P13 | *Unigene5852-RT-F*  *Unigene5852-RT-R*  *Unigene13406-RT-F*  *Unigene13406-RT-R*  *Unigene23140-RT-F*  *Unigene23140-RT-R*  *Unigene25298-RT-F*  *Unigene25298-RT-R*  *Unigene29920-RT-F*  *Unigene29920-RT-R*  *Unigene33285-RT-F*  *Unigene33285-RT-R*  *Unigene34122-RT-F*  *Unigene34122-RT-R*  *Unigene51998-RT-F*  *Unigene51998-RT-R*  *PlActin*-RT-F  *PlActin*-RT-R  *NtActin-RT-F*  *NtActin-RT-R*  *PlPAT1*-F  *PlPAT1*-R  *PlPAT1-TY-F*  *PlPAT1-TY-R*  *PlPAT1*-rbF  *PlPAT1*-rbR | AGTCGCTGCATTACTACTCG  ACAAGCCACCACGTTGCAT  ACACCTTATCCATTAAGCTCCC  CTCCATGCACAAGAGGCAAC  AACCACCCCAAAATCATCCG  TTCCGGCGTCAAAGTACCAGA  CCAATTCAACGGTTAGGTGCATTGGTTCTTTGCACTTTAGAGCTT  TTTCAACAGTAAACCAGCGAGA  GGCTTCAACAAACCTCGGGAA  TCCTCTGTGCTGTTAAAATCCCT  ATCTCCGTGTTCAGATACCAG  CACTGTTTGTACGATATAACTGGG  TGACTAAGGTCCTGTTCAACG  AGATAATCTCTGCCAGTTTGCT  CTCCGTCAATGAATTTGATAGCTT  GTTGCCCTTGATTACGAG  GCTTCCATTCCGATTAGTG  TCCTCATGCAATTCTTCG  ACCTGCCCATCTGGTAAC  ATGACCAATTATTCTTCAATGCAAG  CCTCCATGCACAAGAGGCAAC TTTCATTTGGAGAGAACACCGG  GGGACTCTAGAATGACCAAT  TACAGGACGTAACATAAGGGA  CTGACCACCCGGGTCACCTC  ATACTCGACCCCGGGGGATCCATG  ACCAATTATTCTTCAATGCAAG  GCTCACCATACTAGTTCTAGACCTC  CATGCACAAGAGGCAAC | P1-P10 For qRT-PCR analysis  P11 For full-length cloning of *PlPAT1*  P12 For expression vector construction  P13 For localization vector construction |
