# Supplementary material for: Overexpression of PlPAT1, a GRAS transcription factor from Paeonia lactiflora, enhances salt stress tolerance in transgenic tobacco
Source: BMC Genomics. 2026 Aug 1;27:892. doi: 10.1186/s12864-026-13204-w (PMC13718014; doi:10.1186/s12864-026-13204-w)
Supplement: Supplementary file 3 [file 12864_2026_13204_MOESM3_ESM.docx]

Table S1. Information of GRAS family genes identified in *Paeonia lactiflora*.

| Gene name | Original ID | Length (bp) | AA | GRAS domain | Intron number | Subfamily |
| --- | --- | --- | --- | --- | --- | --- |
| PlDELLA1 | Unigene5852 | - | - | Complete | - | DELLA |
| PlPAT1 | Unigene13406 | 1647 | 548 | Complete | 0 | PAT1 |
| PlSCL8 | Unigene23140 | - | - | Complete | - | PAT1 |
| PlSCL13 | Unigene25298 | - | - | Complete | - | PAT1 |
| PlSCL1 | Unigene29920 | - | - | Complete | - | PAT1 |
| PlSCL4 | Unigene33285 | - | - | Complete | - | SCR |
| PlSCR1 | Unigene34122 | - | - | Complete | - | SCR |
| PlHAM1 | Unigene51998 | - | - | Complete | - | HAM |
